# ASA helps prediction of the death rate in surgical ICU patients

**DOI:** 10.1186/cc13222

**Published:** 2014-03-17

**Authors:** P Batista, R Passos, C Gomes, A Oliveira

**Affiliations:** 1 Hospital Sao Rafael, Salvador Bahia, Brazil

## Introduction

Many scoring systems have been designed to predict mortality in surgical patients; however, these systems require the collection of several parameters that may not be available at ICU admission. As a result, these classification systems are not used as a routine part of clinical practice. The aim of this study was to evaluate the prognostic value of ASA classification predicting ICU and hospital mortality.

## Methods

We analyzed the records of 11,230 ICU admissions. ASA and elective/nonelective nature of the admission were combined into eight categories. We used the chi-square test and accepted the null hypothesis if *P *> 0.05. Areas under the ROC curve using ASA and ASA/ admission categories were constructed.

## Results

We included 5,998 patients admitted to the ICU immediately after surgery. Forty-one percent were older than 65 years and 51% were female. Length of stay previous to ICU admission was 2.5 ± 6.1 days and duration of ICU stay was 1.9 ± 2.4 days. Elective hospital admission occurred in 65% of the patients. Neurosurgery with 21% of the procedures was followed by abdominal surgery (20%), and orthopedic surgery (15%). The death rate at the hospital was 6.9% (2.8% in ASA I patients and 31% in ASA IV/E). Higher ASA classification was significantly associated with the death rate both in the ICU and at the hospital (*P *< 0.00001). The nonelective nature of the hospital admission was also associated with higher risk of death at the ICU (*P *< 0.0001) and in the hospital (*P *< 0.0001). Gender and hour of admission was not associated with the death rate. The combination of ASA classification with the nonelective nature of the hospital admission produced an eight- category index significantly associated with mortality (*P*< 0.00001). Analyzing hospital mortality, the area under the ROC curve was 0.77 (95% CI 0.74 to 0.79) for this index and was 0.72 (95% CI 0.69 to 0.75) for ASA. When analyzed for death at the ICU the AUROC was 0.71 (95% CI 0.69 to 0.75, *P *< 0.0001) for ASA and 0.75 (95% CI 0.71 to 0.78, *P *< 0.0001) for ASA nonelective admission index.

## Conclusion

These results show that the ASA index can be used, preferably in combination with other data from electronic records, to predict mortality for surgical ICU patients.

